# Cell-Derived Vesicles for mRNA Delivery

**DOI:** 10.3390/pharmaceutics14122699

**Published:** 2022-12-02

**Authors:** Zhenghua Li, Zhen Liu, Jiacai Wu, Bin Li

**Affiliations:** 1 Department of Infectious Disease, Shenzhen People’s Hospital, The First Affiliated Hospital of Southern University of Science and Technology & The Second Clinical Medical College of Jinan University, Shenzhen 518020, China; 2 School of Medicine, Southern University of Science and Technology, Shenzhen 518055, China

**Keywords:** cell-derived vesicles, extracellular vesicles, exosome, microvesicles, mRNA delivery

## Abstract

The clinical translation of messenger mRNA (mRNA)-based therapeutics requires safe and effective delivery systems. Although considerable progress has been made on the development of mRNA delivery systems, many challenges, such as the dose-limiting toxicity and specific delivery to extrahepatic tissues, still remain. Cell-derived vesicles, a type of endogenous membranous particle secreted from living cells, can be leveraged to load mRNA during or after their biogenesis. Currently, they have received increasing interest for mRNA delivery due to their natural origin, good biocompatibility, cell-specific tropism, and unique ability to cross physiological barriers. In this review, we provide an overview of recent advances in the naturally occurring mRNA delivery platforms and their biomedical applications. Furthermore, the future perspectives on clinical translation of cell-derived vesicles have been discussed.

## 1. Introduction

Messenger RNA (mRNA) was first discovered in the early 1960s as a critical intermediary between genes and proteins [1]. Since then, significant work has been done towards improving mRNA’s stability and reducing its immunogenicity [2,3,4]. Additionally, the creation of mRNA delivery devices has drawn the attention of numerous researchers [5,6,7,8]. Recently, two coronavirus disease 2019 (COVID-19) mRNA vaccines have just received approval to prevent severe acute respiratory coronavirus 2 (SARS-CoV-2) infection. These progresses have paved the road for mRNA as a new class of drug [2,5,6,7,9,10]. Nowadays, mRNA-based therapeutics have displayed broad applications in prophylactic vaccines, protein replacement therapy, cancer immunotherapy, and gene editing [9,10,11,12,13,14].

It is difficult to exert physiological effects for exogenous mRNA without the assistance of delivery systems, indicating the importance of carriers for mRNA-based therapeutics [5,6,7,8]. Currently, a variety of nanomaterials including lipid- and polymer-based nanoparticles have been developed for mRNA delivery [15,16,17,18]. Among them, lipid nanoparticles are commonly used in preclinical and clinical trials [19,20,21]. Despite the growing number of studies on delivery platforms, many challenges, such as the dose-limiting toxicity and specific delivery of mRNA to extrahepatic tissues, still remain [22,23,24]. In this context, cell-derived vesicles such as extracellular vesicles (EVs) have received increasing attention in the field of mRNA delivery due to their good biocompatibility, cell-specific tropism, and unique ability to cross physiological barriers, such as blood–brain barriers [25,26]. This review summarizes the recent advances in cell-derived vesicle-mediated mRNA delivery and their applications in biomedicine.

## 2. Cell-Derived Vesicles for mRNA Delivery

Cell-derived extracellular vesicles refer to a heterogeneous population of membranous particles that are secreted from living cells, including mammalian cells, bacteria, and fungi [27]. They can be roughly classed into exosomes, microvesicles (MVs), and apoptotic bodies, depending on their biogenesis pathway and size [28]. Exosomes (30–150 nm) are formed as intraluminal vesicles through inward budding of early endosomes and subsequently released into the extracellular space. The plasma membrane directly protrudes outward to generate microvesicles, which range in size from 100 to 1000 nm. When cells die, apoptotic bodies (100–5000 nm) leak out through blebbing plasma membranes. EVs secreted from the source cells (also known as producer cells) transport endogenously produced biomolecules to the nearby recipient cells, or the distant cells through biological fluids [27,29]. Upon entry into the recipient cells, EVs exert important physiological and pathophysiological activity such as modulation of the tumor microenvironment, immunostimulatory functions, and tissue regeneration [29,30,31,32,33,34,35]. Due to their unique biological functions, natural origin, and good biocompatibility, as well as the ability to cross physiological barriers, EVs have attracted great interest as drug delivery platforms for delivery of small molecules (Curcumin, Doxorubicin, Paclitaxel, etc.) and biomacromolecules (siRNA, miRNA, tumor antigen, etc.) [36,37,38,39,40,41,42]. Recently, they have also been leveraged in the field of mRNA delivery and shown promise in multiple therapeutic applications (Table 1) [25]. Other cell-derived vesicles are still relatively unexplored. Until recently, virus-mimicking cell membranes have been reported for coating mRNA-loaded poly (lactic-co-glycolic acid) (PLGA) nanoparticles for enhancing their delivery efficiency, both in vitro and in vivo [43].

### 2.1. Preparation of Cell-Derived Vesicles

Commonly, EVs with or without mRNA cargos should be produced firstly from the producer cells for further isolation and purification. Several types of cells have been explored as source cells of EVs, such as red blood cells, human embryonic kidney 293T cells, bone marrow-derived dendritic cells, and Gram-negative bacteria [58,63,64,65,66]. For EV-based mRNA delivery, 293T were the mostly commonly used cells, accounting for about 80% in all source cells (Table 1). Following secretion, multiple techniques such as ultra-centrifugation, density gradient centrifugation, size exclusion chromatography, and filtration methods have been exploited to obtain purified EVs from source cells [67,68,69,70].

To boost the capability of exosomes production, potential production boosters including STEAP3, syndecan-4, and a fragment of L-aspartate oxidase were employed [51]. STEAP3 and L-aspartate oxidase-related fragment involve the biogenesis and cellular metabolism of exosomes, while syndecan-4 contributes to the inward budding of early endosomes membranes [51]. Co-overexpression of all three boosters in 293T cells significantly increased exosome production [51]. Apart from production boosters, cellular nanoporation (CNP) consisting of a nanochannel array (~500 nm in diameter) has also been proposed for increasing the production of mRNA-loaded exosomes via transient electrical pulses [49]. The yield of mRNA-bearing exosomes for CNP was more than 50-fold higher in comparison with the traditional electroporation method [49]. Furthermore, abundant EVs (10^13^–10^14^ EVs) were produced by treating red blood cells with calcium ionophore [60].

It is worth noting that the heterogeneity of EVs resulted from the process of production and separation has an important impact on their delivery efficiency. Thus, thorough characterization of EVs, including the particle size, zeta potential, morphologies, and surface markers, is necessary for subsequent quality control and biomedical applications [35,67,69,71].

### 2.2. Strategies for mRNA Loading into Cell-Derived Vesicles

**Figure 1:**
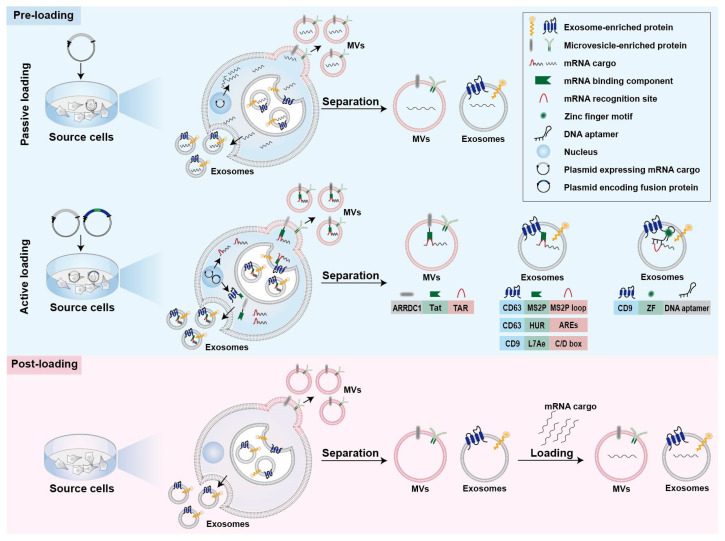
Strategies for mRNA loading into EVs. Strategies are classified into pre-loading methods and post-loading methods. The former can be further divided into passive and active methods. ARRDC1, arrestin domain containing protein 1; Tat, transactivator of transcription protein; TAR, trans-activating response element; MS2P, MS2 bacteriophage coat protein; HUR, human antigen R; MVs, microvesicles; ARE, AU-rich elements; ZF, zinc finger motif.

Strategies for mRNA loading into cell-derived vesicles could be simply classified into two main categories, namely pre-loading methods and post-loading methods (Figure 1) [25,72]. Pre-loading methods (also called pre-separation or endogenous loading methods) heavily rely on the producer cells to pack the mRNA cargos into EVs during their biogenesis (Figure 1) [72]. Sometimes, mRNA-encoded proteins are also simultaneously packaged into cell-derived vesicles during the endogenous loading process [46,48]. Pre-loading methods can be further divided into passive and active categories (Figure 1), as described below [52]. Post-loading methods are also called post-separation or exogenous loading methods (Figure 1) [72]. In this case, exogenous mRNA is loaded into isolated EVs via electroporation or chemical transfection reagents [59,60].

#### 2.2.1. Passive Pre-Loading Methods

The most common approach for passive pre-loading methods is to introduce plasmid into producer cells to obtain the transcribed mRNA of interest. Overexpression of target mRNA could facilitate its enrichment into EVs. Such strategy has been employed by several studies for loading various mRNA into EVs (Table 1) [47,48,50]. For example, low-density lipoprotein receptor (Ldlr) mRNA was encapsulated into exosomes via forced overexpression in the source cells [50]. After plasmid transfection, the level of Ldlr mRNA in source cells increased more than 100-fold compared with cells transfected with control plasmid, thus leading to a similar increase in mRNA cargos in the secreted exosomes [50]. 

Because small RNAs are the dominant modalities of RNAs within secreted exosomes, encapsulation of large mRNA into nano-sized exosomes is technically challenging for passive pre-loading method [25,73]. It is revealed that the aforementioned CNP technology not only increases the yield of exosomes but also improves mRNA content in the exosomes [49]. In comparison with exosomes produced endogenously without external stimulation, the mRNA loading efficiency of CNP-treated exosomes produced by the same source cells increased by three or four orders of magnitude (one mRNA within every 10^3^ exosomes vs. two to ten mRNA per exosome) [49]. Additionally, this strategy also led to a 100-folded higher loading of mRNA into exosomes relative to conventional electroporation method [49].

#### 2.2.2. Active Pre-Loading Methods

Another strategy to improve the loading efficiency of mRNA cargos is to transfect the producer cells with two types of plasmid. One type of the plasmid encodes fusion proteins comprised of mRNA binding components and EV-enriched proteins such as surface markers CD9, CD63, or cytosolic protein Hspa8 (Figure 1) [51,52,53]. Generally speaking, the mRNA of interest transcribed from plasmids contains intentionally engineered recognition sites, which can specifically bind with the mRNA binding components of fusion proteins. The remaining part of the fusion proteins, EV-enriched proteins, are then incorporated into EVs during their biogenesis to achieve active mRNA pre-loading.

Targeted and Modular EV loading (TAMEL) is an active loading platform developed for actively loading mRNA into exosomes via fusing a EV-enriched protein such as Lamp2b, CD63, and Hspa8 to the MS2 bacteriophage coat protein (Figure 1) [52]. The cognate MS2 stem loop sequence was then incorporated into the mRNA cargos to promote mRNA binding and loading into the EVs interior [52]. It has been found that the loading efficiency decreases with the increase in mRNA size [52].

Several other fusion proteins have also been designed for active loading of mRNA into EVs (Figure 1) [51,53]. Archaeal ribosomal protein L7Ae or human antigen R fused to surface marker of EVs are leveraged to bind to the introduced C/D box RNA structure and AU-rich elements in the mRNA cargos, respectively [51,53]. In addition, fusing the transactivator of the transcription protein to the C-terminus of arrestin domain containing protein 1, which mediated the budding of MVs, confers high affinity for binding the stem-loop-containing trans-activating response element introduced at the 5′ end of mRNA cargos [57]. In general, the high binding affinity between mRNA binding components and mRNA recognition site facilitates the active packaging of specific mRNA into EVs.

Apart from these mRNA binding components, DNA aptamer was also used to specifically recognize and actively load the mRNA of interest (Figure 1) [54]. In this case, a specific DNA aptamer consisting of two parts was designed as a bridge for connection between mRNA cargos and EVs [54]. The single strand part of the DNA aptamer could recognize the region surrounding start codon AUG of target mRNA, which was thought to be beneficial for the sorting of mRNA into EVs [54]. The double strand part of the DNA aptamer could be recognized by zinc finger motifs (ZF) that were tailored to specifically bind to the sequence of any double-stranded DNA [54]. To facilitate the recruit of DNA aptamer as well as sorting of the complexed mRNA into EVs, the ZF was fused with an exosomal surface marker, CD9 [54]. As a result, this designed DNA aptamer resulted in a ~2.5-fold increase in the enrichment effect of large PGC1α mRNA into EVs [54].

#### 2.2.3. Post-Loading Methods

So far, post-loading mRNA into EVs largely depends on electroporation and commercial loading reagents (Table 1 and Figure 1). Electroporation is a commonly used method for loading of various molecules, including siRNA and miRNA, as well as mRNA, to purified EVs [60,61,62,74,75]. About one fifth of Cas9 mRNA can be loaded into red blood cell-derived EVs by electroporation [60]. Furthermore, a commercial loading reagent named REG1 has also been used for loading mRNA into EVs after their isolation [59].

### 2.3. Strategies for Tissue-Specific mRNA Expression

**Figure 2:**
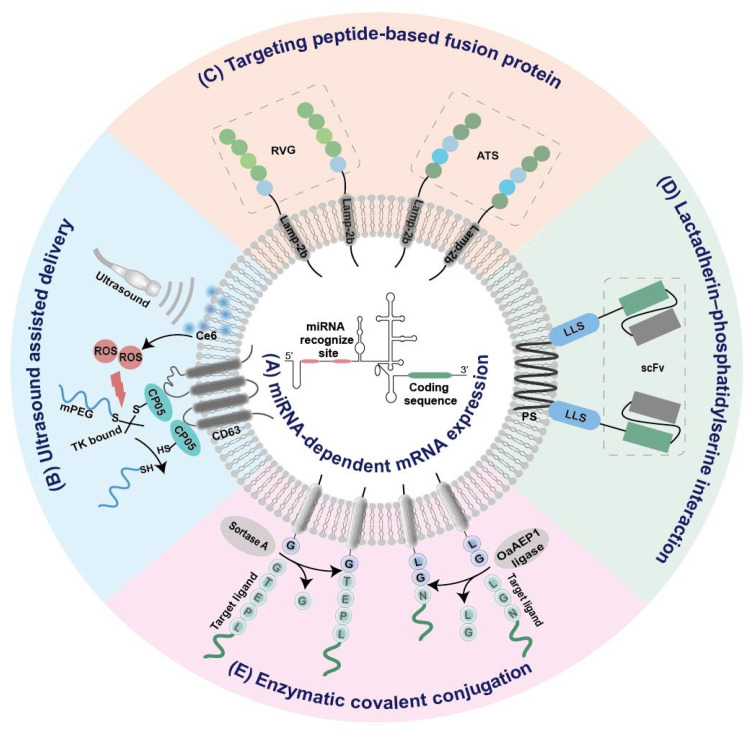
Summary of EV-based platforms for tissue-specific mRNA expression. (**A**) miRNA-dependent mRNA expression. (**B**) Ultrasound assisted delivery. (**C**) Targeting peptide-based fusion protein. (**D**) Lactadherin–phosphatidylserine interaction. (**E**) Enzymatic covalent conjugation. IRES, internal ribosome entry site; RVG, rabies viral glycoprotein; ATS, adipocyte-targeting sequence; scFv, single chain variable fragment; LLS, lactadherin leader sequence; Ce6, sonosensitizer chlorin e6; ROS, reactive oxygen species; PS, phosphatidylserine; TK, thioketal.

To enhance the tissue specificity of cell-derived biomimetic vesicles, several strategies have been proposed, as below (Figure 2).

#### 2.3.1. Tissue-Specific miRNA-Dependent mRNA Expression

It has been found that the internal ribosome entry site (IRES) at the 5′ end of the hepatitis C virus RNA can be specifically recognized by liver-specific miRNA-122, and thus initiates its tissue-specific mRNA translation [76]. Replacement of this miR-122 recognition site at the IRES with sequences recognized by other tissue-specific miRNA enables miRNA-specific activation of mRNA translation in specific tissues (Figure 2A). According to this principle, an adipose-specific translation system (miR-148a-IRES-PGC1α) was constructed by substituting miR-122 recognition sites at the IRES with sequences recognized by adipose-specific miR-148a at the upstream of the *PGC1α* mRNA coding sequence [44]. Injection of exosome loading with such a system resulted in a significant increase in *PGC1α* protein expression in the adipose tissue of mice, but a decrease in lung, spleen, and kidney [44]. Using a similar strategy, the same group also constructed inflammation-responsive Il-10 mRNA by replacing miR-122 with miR-155 enriched in the inflammatory sites of atherosclerosis [47]. The expression of Il-10 mRNA in exosomes was specifically activated by miR-155 in the inflamed macrophages, while its expression in other tissues without obvious inflammation was rare [47].

#### 2.3.2. Ultrasound Assisted Tissue-Specific Delivery

To minimize the off-target effects, exosomal delivery strategies assisted by ultrasound have been explored [44,45]. Recently, two ultrasound-assisted exosomal platforms have been established for the specific delivery of mRNA to adipose tissue [44,45]. In one study, target uptake of EVs was achieved with the assistance of ultrasound-targeted microbubble destruction (UTMD) as well as the tissue-specific miRNA-dependent expression system mentioned above [44]. As the microbubble destruction at the ultrasound site may enhance the cell membrane permeability and cellular uptake of recipient cells, the delivery of exosomes into the adipose tissue should be increased by UTMD. Consistent with this assumption, the distribution of DiR-labeled exosomes in the adipose tissue significantly increased under the assistance of UTMD [44].

In the other study, a smart exosome-based delivery platform was designed for escaping from phagocytosis and locally delivering mRNA to the omental adipose tissue [45]. Firstly, CP05-thioketal (TK)-mPEG was anchored onto exosomes through interaction between the peptide CP05 and the CD63 marker of exosomes (Figure 2B) [45]. The mPEG chain could protect the carrier platform from aggregation, opsonization, and phagocytosis, thus prolonging the in vivo circulation time [45]. Then, reactive oxygen species produced by sonosensitizer chlorin e6 under ultrasound triggered the break of TK bonds between CP05 and mPEG (Figure 2B) [45]. Eventually, the carrier core was exposed by removing the PEG corona and specifically internalized by recipient cells at the ultrasound site, thus leading to a dramatic increase in the expression of exosome-encapsulated mRNA in adipose tissue under ultrasound [45].

#### 2.3.3. Targeted Modification

Conjugation of targeting ligands to EVs via genetic engineering, enzymatic, or affinity-based methods was proven to be effective for exosome-based targeted delivery [77,78,79,80]. For example, when the central nervous system-specific rabies viral glycoprotein (RVG) was fused to an exosomal membrane protein Lamp2b, the fusion protein RVG-Lamp2b facilitated transport of EVs across the blood–brain barrier (Figure 2C) [48,51]. To fulfill the targeted delivery of EVs to adipose tissues, an adipocyte-targeting sequence (ATS, CKGGRAKDC) was also fused to the N-terminus of Lamp2b (Figure 2C) [54]. Moreover, an anti-HER2 single chain variable fragment was connected to a lactadherin leader sequence (Figure 2D) [56]. The former was capable of targeting HER2 overexpressing cells via antigen–antibody interactions, while the latter could bind to EVs based on affinity with their surface phosphatidylserine (Figure 2D) [56]. As a consequence, the modified EVs were selectively internalized by HER2-positive cells [56].

Despite its straightforwardness, the fusion protein-based targeting method always require genetic engineering. Therefore, covalently conjugating peptides or nanobodies onto EVs without any genetic modification of source cells provide an alternative method for the targeted modification of EVs [59]. The versatile targeting platform leverages protein-ligating enzymes (Sortase A and OaAEP1 ligase) to catalyze covalent-bonding reactions between the membrane proteins of EVs and targeting ligands, including targeting peptides, ‘self’ peptides, as well as nanobodies (Figure 2E) [59].

## 3. Application

EVs themselves, or when used as drug delivery systems, are currently being assessed in clinical trials for diagnostic or therapeutic purposes [37]. When functioning as mRNA delivery systems, EVs exhibit a great potential for biomedical applications, including tumor, central nervous system diseases, obesity, and anti-inflammation (Table 1).

### 3.1. Tumor

In 2013, Arda Mizrak et al. pioneered the use of MVs as mRNA carriers for the treatment of tumor [46]. In that study, mRNA encoding cytosine deaminase (CD)-uracil phosphoribosyltransferase (UPRT) fusion proteins were encapsulated into MVs and injected into tumors, in combination with intravenously injected prodrug 5-fluorocytosine (5-FC). The expression of CD and UPRT in tumor cells promoted the conversion of 5-FC to the cytotoxic drug 5-fluoro-deoxyuridine monophosphate, thus inhibiting DNA synthesis and inducing tumor cell apoptosis. The remarkable inhibition of schwannoma tumor growth and regression of tumor size in two animal models indicated the feasibility of MVs as mRNA carrier for tumor therapy [46].

Subsequently, the therapeutic potential of exosomes as mRNA carriers was also explored for tumor therapy [56]. HChrR6 is an optimized bacterial enzyme that could convert prodrug CNOB into cytotoxic drug MCHB. Sequential injection of anti-HER2 scFv antibody-modified HchrR6 mRNA-loaded exosomes and CNOB led to the specific activation of CNOB and subsequent MCHB generation at tumor site, causing near-complete inhibition of orthotopic HER2-positive BT474 xenografts [56]. Additionally, glioma-targeting peptide-modified exosomes carrying PTEN mRNA were applied to brain tumor therapy and increased survival time for U87 or GL261 glioma-bearing mice [49]. 

EVs derived from BL21 (DE3) Escherichia coli with intrinsic function of innate immunity stimulation have also been employed as an mRNA delivery platform for a personalized tumor vaccine [58]. This nanocarrier platform possessed a “Plug-and-Display” feature and allowed the rapid display of various tumor antigens, thus enabling rapid preparation of personalized cancer vaccines. After subcutaneous injection, ovalbumin or ADPGK mRNA delivered by this platform led to significantly strong inhibition of melanoma progression. Moreover, complete regression was observed for three out of eight mice bearing colon cancer [58].

### 3.2. Central Nervous System Diseases

The ability to cross blood–brain barriers makes EVs a promising carrier candidate for the treatment of various brain diseases. Ryosuke Kojima et al. constructed an EXOsomal Transfer Into Cells system (EXOtic) consisting of an exosome production booster, a specific mRNA packaging device, a cytosolic delivery helper, and a brain targeting module (RVG-Lamp2b) [51]. Implantation of exosome producer cells engineered by the EXOtic system into living mice was adopted for the in situ production and delivery of a therapeutic mRNA-containing exosome for treatment of Parkinson’s disease [51]. It has been found that catalase mRNA was delivered into brain tissue by the in situ produced exosomes, resulting in attenuation of neuroinflammation and area-specific rescue of neuronal cell death [51]. Recently, the utility of an EV-based delivery strategy for treatment of cerebral ischemia has also been reported [48]. RVG-modified exosomes loaded with nerve growth factor both in mRNA and protein format were able to reach the ischemic region of the brain following tail vein injection and alleviate ischemic injury by reduced inflammation, improved cell survival, and promoted neurogenesis in ischemia mice [48].

### 3.3. Obesity

Obesity is becoming a health burden worldwide that increases risks for various diseases. EV-mediated mRNA delivery holds promise for obesity therapy [44,45,54]. Two studies have revealed that ultrasound assisted exosome delivery platforms were capable of enhancing the efficacy of functional mRNA at the adipose tissue [44,45]. As both PGC1α (an essential transcription factor for fat browning) and the bone morphology protein 7 (Bmp7, an important inducer of brown adipocyte differentiation) play great roles in the induction of brown adipose tissue, delivery of Bmp7 mRNA or PGC1α mRNA-exosomes to the adipose tissues under ultrasound significantly induced the browning effect, decreased body weight, and reduced off-target effects [44,45]. Furthermore, PGC1α mRNA has been selectively delivered to the adipose via exosomes modified with adipocyte-targeting sequence, eventually leading to a significant decrease in body weight along with an increase in brown adipose tissue [54].

### 3.4. Anti-Inflammation

Il-10, a soluble anti-inflammatory cytokine, plays an important role in inflammation. Thus, Il-10 mRNA-loaded exosomes have been used for the treatment of inflammation related diseases, including atherosclerosis [47] and inflammatory bowel disease [54]. For atherosclerosis, the inflammation responsive Il-10 mRNA-loaded exosomes were efficiently delivered to inflammatory macrophages and precisely translationally activated in inflamed tissues after systemic administration, demonstrating on-demand anti-inflammatory effects with decreased expression of inflammation cytokines, including Il-1β, Tnf-α, and Il-6 [47]. Furthermore, the anti-inflammatory effects also alleviated the atherosclerosis in ApoE-/- mice, with lower atherosclerotic plaques and lesion size [47]. Recently, Il-10 mRNA delivered by exosomes has been reported for the treatment of inflammatory bowel disease [54]. Systemic injection of Il-10 mRNA-loaded exosomes not only reduced inflammatory responses, but also prevented body weight loss and colon length shortening in a mouse model [54].

### 3.5. Other Diseases

Most recently, new attempts to deliver mRNA with cell-derived vesicles have been made in treating other diseases, including familial hypercholesterolemia, acquired immunodeficiency syndrome, and COVID-19 [50,55,62]. Exosomes carrying Ldlr mRNA could restore Ldlr protein expression and further decreased the high serum cholesterol level in Ldlr-/- mice following intravenous injection. Systemic administration of exosomes loaded with ZPAMt mRNA targeting CPG methylation of the 5′ long terminal repeat achieved stably repression of HIV-1 [55]. Furthermore, such exosomes were found to be useful for crossing the blood–brain barrier and inhibiting HIV-1 expression in the brain [55]. More recently, mRNA vaccines against COVID-19 were prepared by electroporation of mRNA encoding SARS-CoV-2 spike into lung-derived exosomes. Such vaccines were able to elicit more potent immune responses than liposome-based counterpart in mice by dry powder inhalation [62].

## 4. Conclusions and Outlook

Cell-derived vesicles hold promise for delivering various small molecules, siRNA, and miRNA. In recent years, these naturally occurring vesicles have been adapted for mRNA delivery. They offer a promising opportunity to enhance the efficacy of mRNA in treating a variety of diseases, ranging from tumor to COVID-19. Nevertheless, some barriers have yet to be solved before translational applications. These include standardization of the critical parameters of inherently heterogeneous EVs, such as surface signatures and internal autologous contents. Furthermore, biodistribution in target organs merits further improvement. Future work on hybrid EVs containing synthetic nanocarriers or the rational design of engineered EVs would provide great value for the development of more effective and selective cell-derived vesicles for mRNA delivery, thus accelerating their clinic translation.

## Figures and Tables

**Table 1:** Cell-derived vesicles for mRNA loading and delivery.

Cell-Derived Nanocarriers	Surface Markers	Size	Source Cell	Loading Strategies	mRNA Cargos	Target Strategies	Application	Ref.
Exosomes	TSG101 and CD9	50–200 nm	293T	Passive loading	Luciferase or PGC1α	miRNA-dependent mRNA expression and ultrasound	Obesity	[44]
Exosomes	CD9, TSG101, and CD63	30–160 nm	293T	Passive loading	Bmp7	Ultrasound	Obesity	[45]
MVs	NA	Mostly 100–150 nm	HEK-293T	Passive loading	CD-UPRT-EGFP	Prodrug	Schwannoma tumor	[46]
Exosomes	TSG101 and CD9	50–200 nm	HEK293T	Passive loading	Il-10	miRNA- dependent mRNA expression	Inflammation of atherosclerosis	[47]
Exosomes	Lamp2b, CD63, Alix, and Tsg101	20–500 nm	HEK293	Passive loading	Nerve growth factor	RVG-Lamp2b	Cerebral ischemia	[48]
Exosomes	CD9, CD63, CD47, and Tsg101	NA	MEFs and DCs	Passive loading based on a cellular nanoporation system	PTEN	Fuse glioma-targeting peptides to CD47	Glioma tumor	[49]
Exosomes	CD9, TSG101	30–150 nm	AML12	Passive loading	Ldlr	NA	Familial hypercholesterolemia	[50]
Exosomes	CD63, Lamp2b, CD9 and TSG101	50–200 nm	HEK-293T	Active loading based on L7Ae-CD63 fusion protein	nluc or catalase	RVG-Lamp2b	Parkinson’s disease	[51]
Exosomes	Lamp2b and CD63	50–200 nm	293FT	TAMEL platform	DTomato or Cas9	NA	NA	[52]
Exosomes	CD63, CD9, and TSG101	100–200 nm	293T	Active loading based on CD9-HUR fusion protein	Cas9	NA	NA	[53]
Exosomes	CD9 and Lamp2b	100–300 nm	293T	Active loading based on a specific DNA aptamer	GFP, PGC1α or Il-10	ATS-Lamp2b	Obesity and intestinal inflammation	[54]
Exosomes	CD63, Alix, and TSG101	Average ~100 nm	HEK293T	Active loading based on L7Ae-CD63 fusion protein	ZPAMt	NA	HIV-1 infection	[55]
Exosomes	CD63, CD81, and lactadherin	30–100 nm;	HEK293	Active loading based on EV-loading zipcode sequence	HChrR6	Prodrug	HER2 + ve human breast cancer	[56]
MVs	ARRDC1	<100 nm	293T	Active loading based on Tat-ARRDC1 fusion protein	GFP or p53	NA	NA	[57]
Outer membrane vesicles	ClyA	average 28.1 nm	BL21 (DE3) Escherichia coli	Active loading based on ClyA-L7Ae fusion protein	EGFP, OVA or ADPGK	NA	Melanoma and colon cancer	[58]
Exosomes	Glycophorin A, ALIX, and TSG1–1	120–200 nm	Red blood cells	Post-loading based on REG1 loading reagent	Luciferase	An enzymatic method	NA	[59]
A mixture of exosomes and MVs	ALIX, TSG101, hemoglobin A, and Stomatin	100–300 nm (average ~140 nm)	Red blood cells	Post-loading based on electroporation	Cas9	NA	NA	[60]
Exosomes	CD63	~110 nm	HEK293T or lung spheroid cells	Post-loading based on electroporation	GFP	NA	NA	[61]
Exosomes	NA	~200 nm	HEK293T or lung spheroid cells	Post-loading based on electroporation	GFP or SARS-CoV-2 spike protein	NA	COVID-19	[62]
Cell membrane-coated PLGA NPs	NA	average 185 nm	B16F10	Double emulsion method with the assistance of G0-C14	EGFP or Cypridina luciferase	NA	NA	[43]

MEFs, mouse embryonic fibroblasts; DCs, dendritic cells; HUR, human antigen R; ARRDC1, arrestin domain containing protein; Cas9, CRISPR-associated protein 9; GFP, green fluorescent protein; nluc, NanoLuc luciferase; EGFP, enhanced green fluorescent protein; OVA, ovalbumin; Ldlr, low density lipoprotein receptor; RVG, rabies viral glycoprotein. PTEN, a tumor suppressor phosphatase and tensin homolog deleted on chromosome 10; Lamp2b, lysosome-associated membrane protein 2; SARS-CoV-2, severe acute respiratory coronavirus 2; COVID-19, coronavirus disease 2019; NA, not applicable.

## Data Availability

Not applicable.
